# Management of Acute Appendicitis in Children During COVID-19 and Perspectives of Pediatric Surgeons From South Asia: Survey Study

**DOI:** 10.2196/26613

**Published:** 2021-12-21

**Authors:** Md Jafrul Hannan, Mosammat Kohinoor Parveen, Md Mozammel Hoque, Tanvir Kabir Chowdhury, Md Samiul Hasan, Alak Nandy

**Affiliations:** 1 Department of Pediatric Surgery South Point Hospital Chittagong Bangladesh; 2 Department of Pharmacology Rangamati Medical College Rangamati Bangladesh; 3 Department of Pediatric Surgery Chattagram Maa-O-Shishu Hospital Medical College Chittagong Bangladesh; 4 Department of Pediatric Surgery Chittagong Medical College Chittagong Bangladesh; 5 Department of Pediatric Surgery Dhaka Shishu Hospital Dhaka Bangladesh; 6 Department of Anesthesiology Chattgram Maa-O-Shishu Hospital Medical College Chittagong Bangladesh

**Keywords:** COVID-19, gastrointestinal, pediatric, global surgery

## Abstract

**Background:**

Nonoperative treatment (NOT) of pediatric appendicitis as opposed to surgery elicits great debate and is potentially influenced by physician preferences. Owing to the effects of the COVID-19 pandemic on health care, the practice of NOT has generally increased by necessity and may, in a post–COVID-19 world, change surgeons’ perceptions of NOT.

**Objective:**

The aim of this study was to determine whether the use of NOT has increased in South Asia and whether these levels of practice would be sustained after the pandemic subsides.

**Methods:**

A survey was conducted among pediatric surgeons regarding their position, institute, and country; the number of appendicitis cases they managed; and their mode of treatment between identical time periods in 2019 and 2020 (April 1 to August 31). The survey also directly posed the question as to whether they would continue with the COVID-19–imposed level of NOT after the effect of the pandemic diminishes.

**Results:**

A total of 134 responses were collected out of 200 (67.0%). A significant increase in the practice of NOT was observed for the entire cohort, although no effect was observed when grouped by country or institute. When grouped by position, senior physicians increased the practice of NOT the most, while junior physicians reported the least change. The data suggest that only professors would be inclined to maintain the COVID-19–level of NOT practice after the pandemic.

**Conclusions:**

Increased practice of NOT during the COVID-19 pandemic was observed in South Asia, particularly by senior surgeons. Only professors appeared inclined to consider maintaining this increased level of practice in the post–COVID-19 world.

## Introduction

Nonoperative treatment (NOT) of acute uncomplicated appendicitis (AUA) refers to treatments involving prescription of antibiotics only, typically those with aerobic and anaerobic coverage of common bacteria commonly found in the bowel. Examples include treatments with amoxicillin and clavulanic acid, piperacillin sodium and tazobactam sodium, or ciprofloxacin hydrochloride and metronidazole hydrochloride. In the Appendicitis Acuta (APPAC) randomized controlled trial [1], intravenous ertapenem was administered for 3 days, followed by 7 days of oral levofloxacin and metronidazole. A combination of intravenous ceftriaxone and metronidazole was used in another study [2].

In South Asian countries, AUA is diagnosed using clinical features, physical examinations, total blood count, and ultrasonograms during outpatient checkups. Complicated appendicitis is considered if there is evidence of appendix perforation, abscess formation, localized peritonitis, or lump formation on clinical examinations supported by ultrasonogram. NOT is considered if diagnosis was clinically suspected as appendicitis plus leukocytosis, with confirmation by ultrasonogram. Patients are asked to report to the outpatient department after 7 days of treatment or to the emergency department in case of worsening conditions. NOT is abandoned when no improvement with antibiotics, clinical deterioration, and development of signs and symptoms of complicated appendicitis are observed.

However, treating pediatric acute appendicitis nonoperatively, rather than by open or laparoscopic appendectomy, still elicits great debate [3-5] spawned by the results of controlled trials and observational studies [6-10], reviews [11-14], and meta-analyses [15-17]. The year 2020 has provided a unique opportunity in which some of this opinion bias, particularly against NOT, can be partially removed. In a recent editorial, the pandemic was considered to provide an opportunity for the ultimate trial for NOT [18]. COVID-19, which is caused by SARS-CoV-2, emerged from Wuhan, China, in December 2019. As of November 2021, the infection has spread globally, with over 250 million confirmed cases resulting in over 5 million deaths worldwide [19]. The response to the pandemic has affected all social and economic aspects of life. However, it has also significantly affected health care facilities [20] in several ways, such as reallocation of hospital facilities to accommodate the influx of COVID-19 patients as well as reducing the number of procedures that would normally be carried out as a result of the repurposing of rooms and maintaining of social distancing protocols to mitigate the spread of the virus. Nearly all countries (90%) have experienced such disruptions to their health services, with low- and middle-income countries reporting the greatest difficulties [20]. This effect has particularly strained surgeries, in general [20,21], and, thus, would have a direct effect on treatment strategies for pediatric AUA. The objective of this study was to determine the effect of the COVID-19 pandemic on the approaches of care for AUA in the South Asian countries of Bangladesh, India, Nepal, and Pakistan, and to determine if any permanent changes in opinions of care will result once the effects of the pandemic have diminished.

## Methods

The instrument used in this study was a voluntary survey with no added incentives; the survey was administered via SurveyMonkey (Momentive), a leading provider of survey software and tools, and was sent to 200 pediatric surgeons in Bangladesh, India, Pakistan, and Nepal. The invitation to participate was sent via email, mobile SMS, Facebook Messenger, and WhatsApp to physicians on mailing lists and to physicians in all coauthors’ personal networks. In the invitation, prospective respondents were told the length of time the survey would be available, which data would be stored, the data storage location, and for how long data would be kept. No personal information was collected. The purpose of the study was explained, and the investigators were identified. The open survey consisted of 10 nonrandomized questions pertaining to the respondents’ position, type of institute, and country of practice; the number of appendicitis cases they managed between April 1 and August 31 in 2019 and 2020; and the number and percentage of those cases that were treated nonoperatively—responses were binned within 10% increments. Respondents were able to change their responses prior to submission and were also alerted to any missed or skipped questions. Data were collected over a 2-week period from September 14 to 28, 2020; each participant was given a unique anonymous identifier. The survey questions were conceived and developed by all coauthors, but no statistical validation was performed. The Checklist for Reporting Results of Internet E-Surveys (CHERRIES) checklist [22] for the survey is included in Multimedia Appendix 1. The final question posed to each pediatric surgeon was whether they would maintain their current COVID-19–imposed level of NOT once the effect of the pandemic on their ability to perform surgery subsides. The study was approved by the Ethics Committee of South Point Hospital, Chittagong, Bangladesh (No. Admn/SPH/190/2020).

The survey response data were analyzed using JMP software (version 15.2; SAS Institute Inc). There was no weighting of responses assigned to certain questions. We use matched-pair tests for each respondent and analyzed the results using a Wilcoxon signed-rank test. The analyses were performed ungrouped and grouped by country, type of institute, and position.

## Results

A total of 134 out of 200 physicians responded to the survey, representing a 67.0% response rate. There were no missing data. Respondents’ positions, types of institute, and countries of practice are summarized in Table 1.

**Table 1:** Descriptive statistics of the survey participants’ responses regarding position, hospital type, and country of practice.

Participant information		Participants (N=134), n (%)
**Position**		
	Resident	14 (10.4)
	Registrar or assistant registrar	21 (15.7)
	Assistant professor	26 (19.4)
	Associate professor	29 (21.6)
	Professor	20 (14.9)
	Consultant	24 (17.9)
**Type of institute**		
	Private practice	11 (8.2)
	Private medical college	35 (26.1)
	Medical university	12 (9.0)
	Government medical college	44 (32.8)
	Corporate hospital	9 (6.7)
	Children’s hospital	23 (17.2)
**Country of practice**		
	Pakistan	28 (20.9)
	Nepal	6 (4.5)
	India	27 (20.1)
	Bangladesh	73 (54.5)

**Figure 1:**
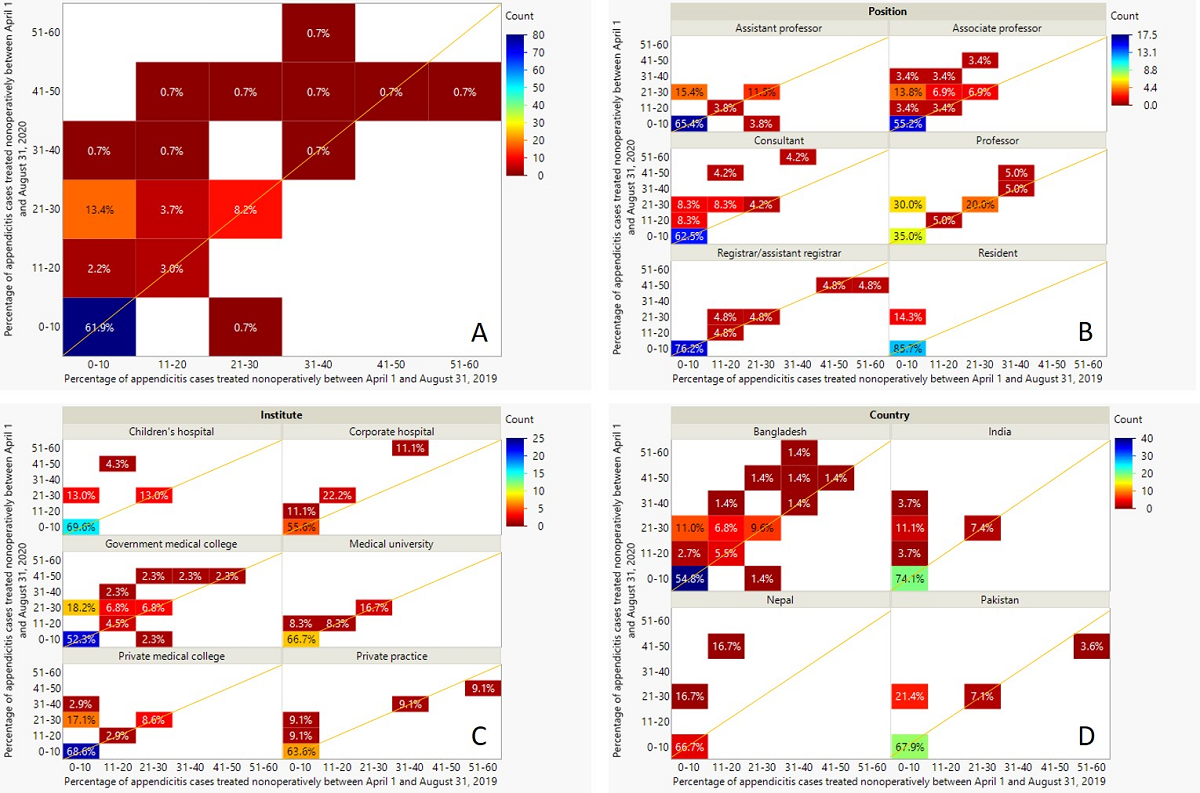
Heat maps depicting the change in usage of nonoperative treatment (NOT) by each physician in the entire cohort (A) and grouped by position (B), institution type (C), and country (D). Boxes entirely above the diagonal indicate an increase in usage of NOT. The values in each box represent the percentage of physicians (N=134).

The change in usage of NOT by each physician in the entire cohort, as well as grouped by position, institution type, and country, is depicted by the heat maps in Figure 1, A to D, respectively. The values in each box represent the percentage of physicians. Boxes that lie completely above the diagonal represent physicians who increased their usage of NOT during the pandemic. The results from the Wilcoxon signed-rank tests are given in Table 2 for the entire cohort as well as grouped by position, institute, and country. There was a significant increase in the percentage of appendicitis cases treated nonoperatively from April 1 to August 31, 2020, compared to the same time period in 2019 (*P*<.001), as clearly observed in Figure 1, A. Though it appears there may be some differences when the cohort is grouped by type of institute or country (Figure 1, C and D), these were not found to be significant (*P*=.65 within pairs and *P*=.52 among pairs for type of institute; *P*=.65 within pairs and *P*=.52 among pairs for country). When grouping by position, we found a weak significance within pairs (*P*=.06), which compares the differences between pairs in a group, but not among pairs (*P*=.12), which compares the averages of the pairs between groups.

**Table 2:** Summary of statistics from the matched-pair analysis comparing percentage of appendicitis cases receiving nonoperative treatment in 2019 and 2020 for the entire cohort and the grouped by position, institute, and country.

Samples analyzed		*P* value^a^
Entire cohort		<.001
**Within pairs**		
	Grouped by position	.06
	Grouped by institute	.65
	Grouped by country	.65
**Among pairs**		
	Grouped by position	.12
	Grouped by institute	.52
	Grouped by country	.25

^a^*P* values were based on Wilcoxon signed-rank tests comparing 2019 and 2020 values.

**Figure 2:**
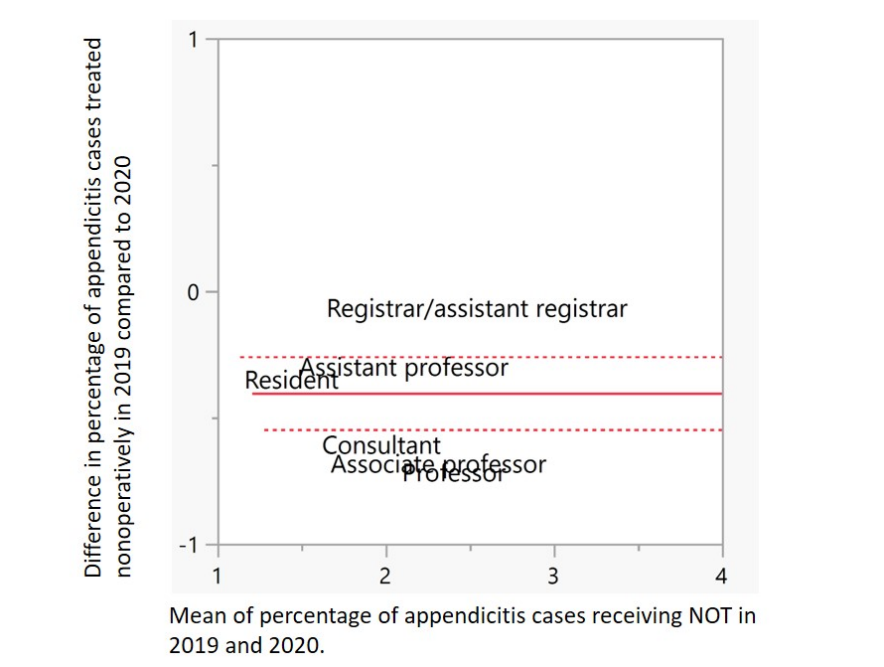
Matched-pair plot for positions showing the difference between pairs plotted against the average between pairs. The time periods in 2019 and 2020 are April 1 to August 31. The red dotted lines indicate the boundaries of the 95% CI and the solid line represents the mean for the entire cohort. NOT: nonoperative treatment.

This potentially interesting effect of the position grouping is shown in more detail in the matched-pair plot in Figure 2. As this is grouped data, the y-axis reports the mean of the differences between the groups, and the x-axis represents the mean of the means. For clarity, the data points have been removed and only the group labels are plotted. The dotted red lines indicate the boundaries of the 95% CI, and the solid line represents the mean for the entire cohort. It should be noted that the upper 95% CI value falls below zero. Not only does this plot clearly show that the percentage of appendicitis cases treated nonoperatively has significantly increased in 2020, but it also indicates that professors, consultants, and associate professors were most likely to expand their use of NOT. This is also evident from Figure 1, B.

**Figure 3:**
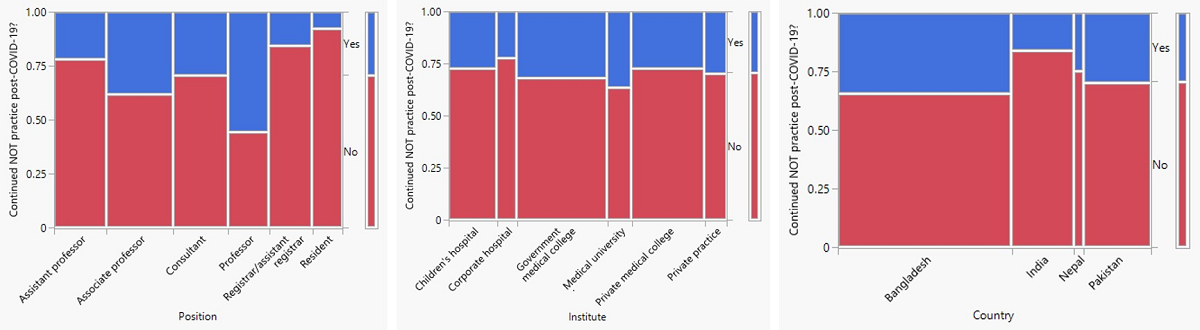
Mosaic plots illustrating the distribution of responses to the question of adopting nonoperative treatment (NOT) for appendicitis after the effects of the pandemic on surgery capability subside, between April 1 and August 31, 2020.

Also interesting are the results from the final question as to whether or not the physicians would consider maintaining the COVID-19–imposed level of NOT for appendicitis after the effects of the pandemic on surgery capability subside. An interesting result from this points to the level and spirit of opinion on the topic of NOT, in general. Though the respondents were given the option of replying “don’t know” or “no comment,” the majority (126/134, 94.0%) responded with a definitive “yes” or “no.” The fractions of those grouped by position, institute, and country are shown in the mosaic plots in Figure 3. As in the matched-pair analysis, no association was found using the Fisher exact test between institute (*P*=.99) or country (*P*=.37) and a planned change in the level of NOT practice after the effects of the pandemic subside. However, a significant association was found for position (*P*=.04). The mosaic plot in Figure 3 shows that residents were the least likely, and professors the most likely, to retain their current level of NOT postpandemic.

The data sets generated and analyzed during this study are available online [23].

## Discussion

### Principal Findings

The results from this study indicate that, despite being required to be more reliant on the usage of NOT for pediatric appendicitis as necessitated by the effects of the COVID-19 pandemic on health care, it has done little to sway the opinion of most physicians in Bangladesh, Nepal, India, and Pakistan regarding its place in the treatment of AUA. Only those practicing with appointed professorships have appeared to give the idea of making the increased usage of NOT a permanent practice after the effects of the pandemic wane. The results of this study indicated an increased usage of NOT, particularly by professors, associate professors, and consultants. This is in good agreement with a recent study by Irish surgeons, where 76% of the 161 participants disclosed that they had modified their practice to a predominantly NOT approach [24]. Similarly, in a New York hospital that traditionally favors operative management, NOT usage increased to roughly 50% of all cases as a result of the pandemic, with favorable results [25]. In an Indian study, Verma et al [26] documented how their institute increased their percentage of NOT to 69% of patients, a significant increase from 22% recorded during the same time period in 2019. The recent work of Ielpo et al [27] also showed an increased usage of NOT in pediatric appendicitis during the pandemic, as did a study from the United Kingdom [28]. More recently, the usage of NOT and the resulting outcomes of AUA in the setting of COVID-19 was systematically reviewed. The results indicated that NOT was a safe, short-term alternative to surgery and led to acceptably low rates of failure and complication [29]. However, a global study on the effect of COVID-19 on pediatric surgery, in general, showed less of an effect, where only 4 out of 20 institutes were found to begin using NOT for appendicitis, and 14 out of 20 reported that they had not changed their extent of NOT use [30].

Another interesting observation coming from this study relates to the results from Pakistan. During the pandemic, there was a clear mandate to change all procedures in children that showed no clinical or radiological signs of complicated appendicitis to a treatment regimen of antibiotics administered intravenously [31]. Yet, the results of this study (eg, Figure 1, D) do not reflect this policy particularly well unless the majority of cases were overwhelmingly complicated appendicitis cases. This may be due to lockdown-related delays in hospital visits of children with acute appendicitis that spawned an increased rate of complicated appendicitis, as documented in a Spanish study [32].

The results of this study indicated that junior physicians were more likely to retain their prepandemic rate of operative treatment compared to their more senior counterparts. While the reason for this is currently unknown, the survey results indicate that 79% of those who identified themselves as residents came from government medical colleges and universities. It might be plausible that the reason for their reluctance to convert to NOT may be a result of the confounding factor that physicians at these institutions are also under pressure to simultaneously maintain their level of education, particularly since there is, in general, a shortage of surgeons in these regions. This would provide a driving force for them to gain experience and continue with surgical techniques.

In all previous studies on the treatment of AUA amid the COVID-19 pandemic, little, if anything, is discussed about how the physicians would modify their practice of NOT once the crisis is over. This study directly addressed this question and the results indicated that it is mainly professors who would consider adapting to change, and residents would be the least likely. The residents would have been in the early stages of the learning curve of surgical techniques, be it open or laparoscopic, and may have had less experience with NOT regimens and the diagnostics required for their safe recommendation. Based on this, it would be understandable that they would not be keen on moving more toward NOT methods when they are in the midst of mastering their surgical skills.

A limitation of this study, which is independent of the physicians’ personal opinions or preferences, is that the resources and capacities of hospitals will be different postpandemic, a factor that was not measured in the survey. This could also sway the usage of NOT toward those facilities with the scarcest of capacities and resources. Ultimately, all of these factors can and should contribute to treatment decisions [33]. Even when the effects of the pandemic subside, the economic effects will lag considerably, and recovery will be slow. This will, in turn, continue to place economic pressures on institutions, which may necessitate longer-than-anticipated usage of NOT for AUA. Another limitation of this study was that the survey was not validated in any formal manner, as it was designed as more of an information gathering tool. Selection bias may be a factor, though the use of both personal networks from all coauthors, rather than just one, and mailing lists used to reach out to potential candidates helped to alleviate this effect.

### Conclusions

The global COVID-19 pandemic has resulted in severe disruption of surgeries worldwide; this has necessitated new approaches to maintain care for those in need, when so many resources are being repurposed to address the massive influx of patients stricken by the virus. In the specific area of pediatric appendicitis, there is ample evidence that more hospitals and institutes are increasing their implementation of NOT for AUA in Bangladesh, India, Pakistan, and Nepal. In some respects, pediatric surgeons could think of this as their participation in an involuntary clinical trial. The results suggest that only professors in these countries would consider maintaining this increased level of practice in the post–COVID-19 world and that the effect of institution type or country was insignificant. It is likely that the demands of continuing education of the younger cohort contributes to their reluctance to practice more NOT postpandemic, just as they are beginning the climb the learning curve for operative treatments.

## Appendix

Multimedia Appendix 1 Checklist for Reporting Results of Internet E-Surveys (CHERRIES).

## Abbreviations

APPAC: Appendicitis Acuta
AUA: acute uncomplicated appendicitis
CHERRIES: Checklist for Reporting Results of Internet E-Surveys
NOT: nonoperative treatment
